# Formation of GeO_2_ under Graphene on Ge(001)/Si(001) Substrates Using Water Vapor

**DOI:** 10.3390/molecules27113636

**Published:** 2022-06-06

**Authors:** Ewa Dumiszewska, Paweł Ciepielewski, Piotr A. Caban, Iwona Jóźwik, Jaroslaw Gaca, Jacek M. Baranowski

**Affiliations:** Łukasiewicz Research Network, Institute of Microelectronics and Photonics, Al. Lotnikow 32/46 Str., 02-668 Warsaw, Poland; pawel.ciepielewski@imif.lukasiewicz.gov.pl (P.C.); piotr.caban@imif.lukasiewicz.gov.pl (P.A.C.); iwona.jozwik@imif.lukasiewicz.gov.pl (I.J.); jaroslaw.gaca@imif.lukasiewicz.gov.pl (J.G.); jacek.baranowski@imif.lukasiewicz.gov.pl (J.M.B.)

**Keywords:** graphene, Raman spectroscopy, germanium oxide

## Abstract

The problem of graphene protection of Ge surfaces against oxidation is investigated. Raman, X-Ray diffraction (XRD), atomic force microscopy (AFM) and scanning electron microscopy (SEM) measurements of graphene epitaxially grown on Ge(001)/Si(001) substrates are presented. It is shown that the penetration of water vapor through graphene defects on Gr/Ge(001)/Si(001) samples leads to the oxidation of germanium, forming GeO_2_. The presence of trigonal GeO_2_ under graphene was identified by Raman and XRD measurements. The oxidation of Ge leads to the formation of blisters under the graphene layer. It is suggested that oxidation of Ge is connected with the dissociation of water molecules and penetration of OH molecules or O to the Ge surface. It has also been found that the formation of blisters of GeO_2_ leads to a dramatic increase in the intensity of the graphene Raman spectrum. The increase in the Raman signal intensity is most likely due to the screening of graphene by GeO_2_ from the Ge(001) surface.

## 1. Introduction

The question of protection of various surfaces by graphene is quite open. Due to the tight graphene lattice, it may be expected that it should protect surfaces against oxidation well. In this work protection of germanium surfaces by graphene was investigated. The CVD growth of graphene on Ge(001)/Si(001) substrates has been used for this purpose. This method of growth was proposed as a promising method for obtaining graphene layers on large area substrates [1,2,3,4,5,6,7,8,9,10,11]. It has been shown that depending on the germanium face and growth conditions, it is possible to obtain graphene nanoribbons [8] and even high-quality graphene layers [9]. It was also pointed out that the growth of graphene on top of Ge(001)/Si(001) substrates is important [3,7,11]. There have been efforts to study intercalation and oxidation of the buried graphene-Ge interface by chemical vapor [12] and by H_2_ [13]. Therefore, the issue of oxidation of the graphene-Ge interface deserves comprehensive investigation.

The graphene growth on Ge(001)/Si(001) substrates is complicated due to the formation of germanium nano-facets underneath the graphene [11,14]. The hill-and-valley structures on the Ge surface are positioned 90° to each other and run along the <100> directions. Graphene nucleation reproduces the Ge(001) nano-faceting and creates multidomain graphene formation. The most likely explanation is that the growth originates on the terraces of the hill structures, which are well-oriented in the <100> direction [11]. That leads to different orientational discontinuities in the graphene layer grown. Therefore, due to the perpendicular orientation of the hill-and-valley structures, the merging graphene layer has a polycrystalline character. This is confirmed by LEED measurements, which indicate that two orientations within the graphene layer are observed [11]. The boundaries between the two oriented domains are the origin of the unintentional oxidation, as shown by Kelvin probe force microscopy (KPFM) together with scanning tunneling microscopy/spectroscopy (STM/STS) studies [15]. The oxidation of Ge(001) and Ge(110) surfaces has only recently been reported [16]. It was argued that the oxidation takes place through atomic-sized openings in the graphene domain boundaries. The penetration of water under the graphene grown on the Ge(001) surface has also recently been reported [17]. A fast oxidation process through 3 μm of germanium in Gr/Ge(001)/Si(001) samples by the penetration of water under light illumination has also recently been reported [17]. In our work the oxidation through the most likely domain boundaries in graphene in Gr/Ge(001)/Si(001) samples is investigated. However, the novelty of our work relies on the observation that for oxidation of germanium surfaces, various graphene defects are responsible. It seems that dissociation of water molecules on graphene defects is energetically favorable.

## 2. Methods and Results

Graphene films were synthesized in a 6-inch Aixtron Black Magic system using the CVD method on Ge(001)/Si(001) wafers which contained 3 μm thick Ge film. During the graphene deposition a pressure of 850 mbar was sustained, and the temperature was 900 °C. Methane gas mixed with Ar in the ratio of 1:200 was used as a carbon precursor. The CVD growth was performed on 2 inch wafers preceded by annealing the substrate under pure hydrogen to reduce native oxides.

A scanning electron microscope (SEM) (Auriga CrossBeam Workstation, Carl Zeiss, Jena, Germany) equipped with a secondary-electron detector was used to observe the influence of water vapor on the graphene morphology.

Room temperature Raman measurements were done using a Renishaw Via Raman micro-scope system with a 532 nm Nd:YAG laser as an excitation source, with a laser spot diameter of approximately 0.5 μm. The laser was focused on the sample using a ×100 objective and a numerical aperture NA = 0.9 with the average power of the laser being about 5 mW. Spatial Raman maps were performed in the area of 10 μm × 10 μm (15) with a distance of 1 micron between collected spectra. The Raman peaks were fitted with a single Lorentzian/Gaussian shape using the Renishaw WiRE.4.0 program (Wotton-under-Edge, UK).

The atomic force microscopy (AFM) was measured on a Veeco Dimension V SPM with a NanoScope V controller. The surface images were flattened and plain fitted using Nanoscope analysis software. OTESPA Al coated probes with a constant force of ~42 N/m with a typical nominal tip radius of 7 nm were used for the AFM imaging. All the AFM measurements were performed in a normal atmosphere at room temperature.

The measurement of the XRD curve was performed using a SmartLab diffractometer equipped with a 9-kV rotating Cu-anode.

**Figure 1:**
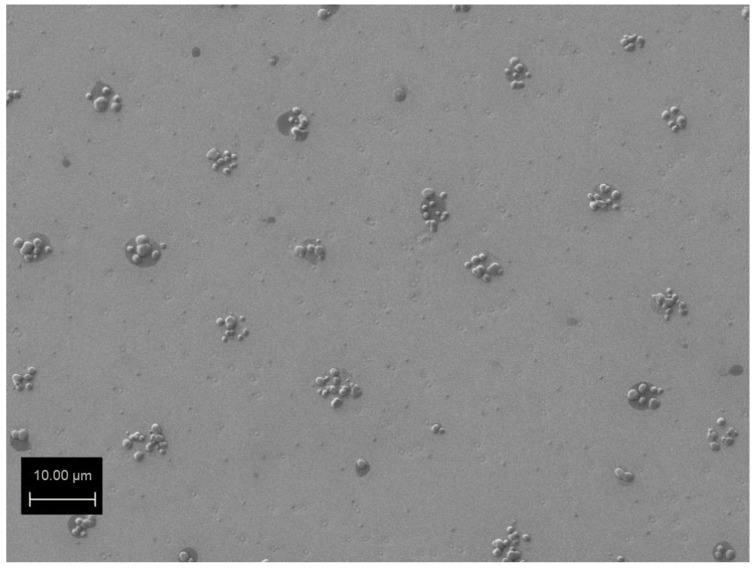
SEM image of the surface of the Gr/Ge(001)/Si(001) after 3 h of water vapor treatment.

**Figure 2:**
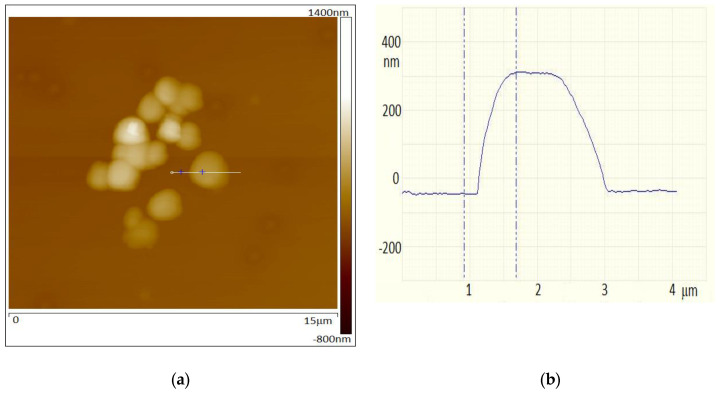
(**a**) AFM image of a group of spots on the surface of the Gr/Ge(001)/Si(001) sample after 3 h of water vapor treatment (**b**) AFM scan of one of the blisters showing that its surface is lifted up compared to the Ge(001) level.

After growth the 1 cm × 1 cm samples were subjected to a humid atmosphere (about 90%) at 50 °C for about 3 h or about 150 h. The samples were placed in a heated container filled with water upon the water surface. The temperature and humidity were measured in the simplest way using a thermometer and a hygrometer. After 3 h of water vapor treatment it was possible to observe regular spots of the size of a few micrometers on the surface of the Gr/Ge/Si(001) sample on the SEM. The SEM image of this surface is shown in Figure 1. The spots consist of groups of smaller blisters which are located at distances of about 10–20 nm. The density of the blisters on the surface presented in Figure 1 is close to 3 × 10^8^ cm^−2^. The AFM image of one group of blisters is shown in Figure 2a. An AFM scan of one of the blisters is shown in Figure 2b. It can be seen that the surface of the blister is about 300 nm higher than its surroundings. It may be expected that the graphene resting on a blister such as this will be under tensile strain.

**Figure 3:**
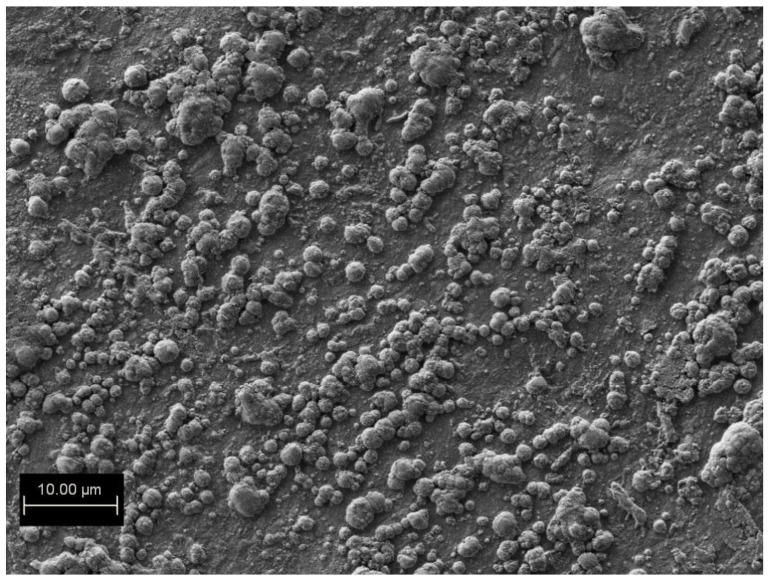
SEM image of the Gr/Ge/Si(001) surface after 150 h of water vapor treatment.

**Figure 4:**
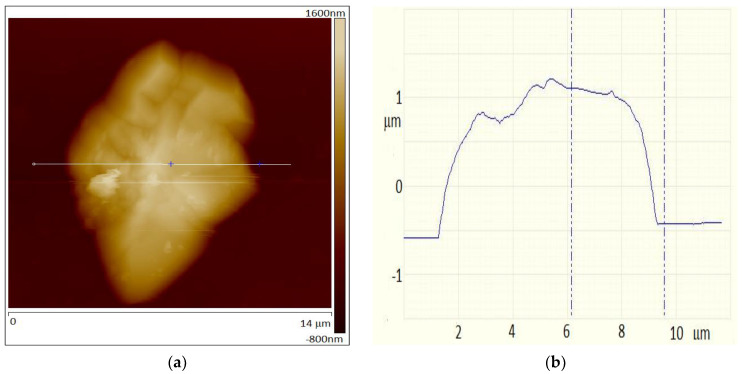
(**a**) AFM image of a blister on the surface of the Gr/Ge/Si(001) after 150 h of treatment in water vapor, (**b**) AFM scan of the blister.

The longer water vapor treatment, up to 150 h, dramatically changed the morphology of the surface of the Gr/Ge/Si(001). The SEM image of the surface is shown in Figure 3. The whole surface is now covered in blisters. The AFM image of one of these blisters is shown in Figure 4a. An AFM scan of the blister is shown in Figure 4b. It can be seen that the blister consists of smaller ones which have merged together. The height of the blister is about 1.5 micrometer.

**Figure 5:**
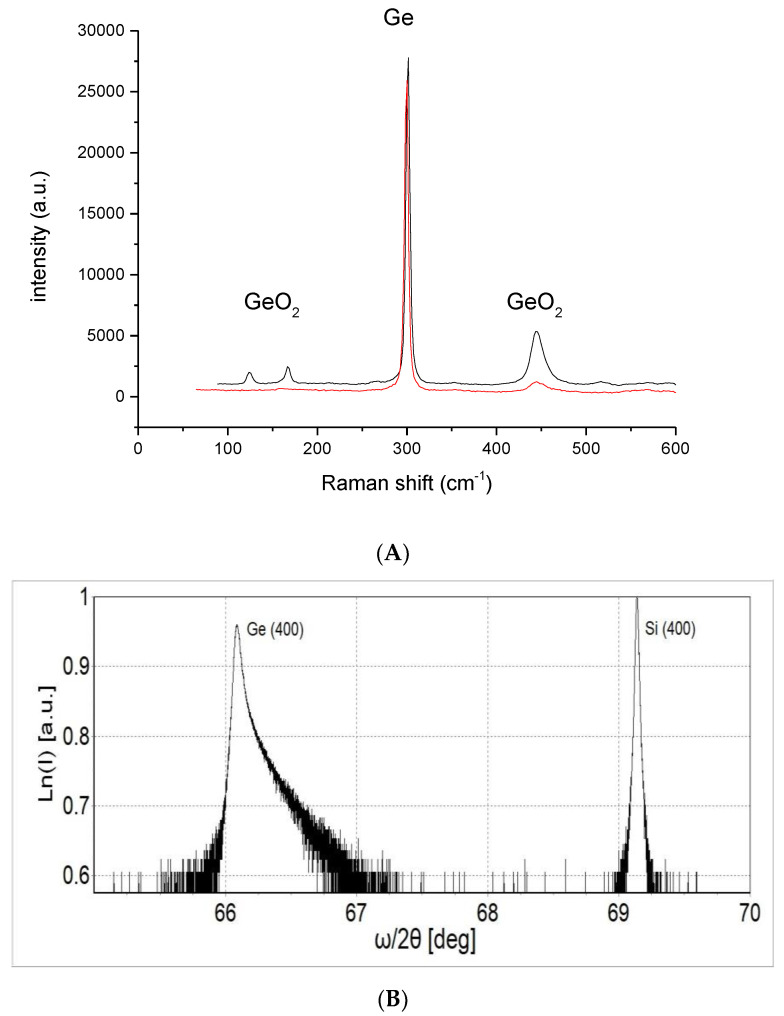
Identification of germanium oxide by (**A**) Raman spectroscopy and (**B**) XRD. Germanium oxide and germanium peaks observed on the blister on the surface of Gr/Ge(001)/Si(001). The bottom (red) and upper (black) lines correspond to the samples after 3 and 150 h of water vapor treatment, respectively.

We wondered what the origin of the blisters on the surface of the Gr/Ge/Si(001) was. The Raman measurements indicate that in the blister locations, for both samples after 3 and 150 h of water vapor treatment, in addition to the intense Ge mode, the spectrum characteristic for germanium oxide occurs. The observed lines at 444 cm^−1^, 166 cm^−1^ and 123 cm^−1^ shown in Figure 5 are characteristic for trigonal GeO_2_ [18]. This spectrum is observed only on the blister area and does not occur outside of this area. The intensity of the GeO_2_ Raman modes appears approximately proportional to the thickness of the blisters. The fact that GeO_2_ blisters already appear after 3 h of water vapor treatment indicates that the influence of water vapor strongly speeds up the oxidation process of Ge. The samples kept in the “drawer” (room temperature and low humidity atmosphere) have not shown any occurrence of GeO_2_. Additionally, there is no sign in the spectra of Raman Si mode (520 cm^−1^), which indicates that the Ge layer is not completely dissolved under the blisters. As can be seen from the lower part of Figure 5, the (400) germanium reflection is asymmetric on the higher angle side, which means that at least part of the Ge layer has a lattice constant smaller than the standard 5.6461 Å. This may be explained by contamination of the germanium by the presence of the trigonal germanium dioxide phase, whose lattice parameters are smaller than those of germanium (a = 4.9021 Å, c = 5.5708 Å) [19]. The presence of the trigonal germanium dioxide phase is confirmed by the Raman measurement.

**Figure 6:**
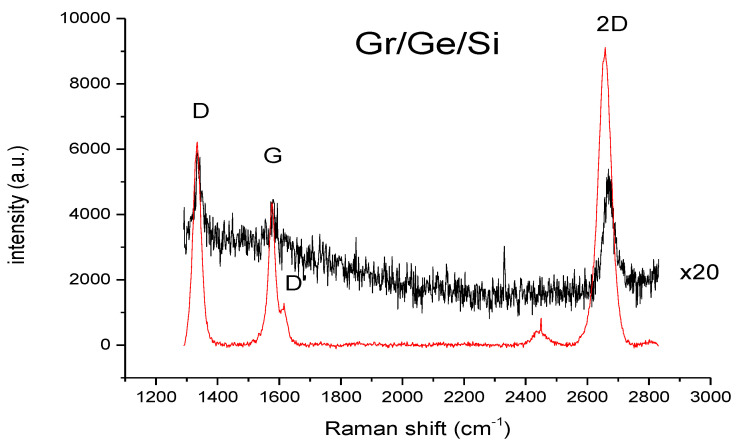
Raman spectra on Gr/Ge/Si(001) (black line) outside the blister multiplied by a factor of 20, and the one on the blister for maximal enhancement (red line).

The intensities of the Raman spectra of graphene are quite different in the region of blisters versus further away from them. The corresponding spectra are shown in Figure 6. The spectrum for the virgin sample is the same as the one shown for outside the blister in Figure 6.

**Figure 7:**
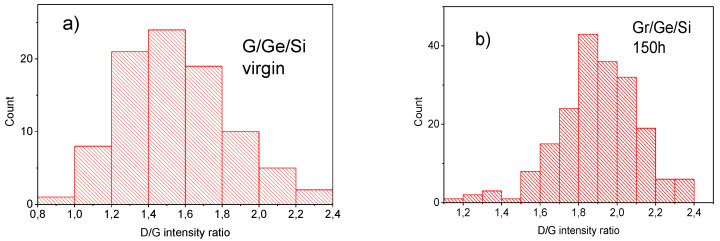
Histograms of the intensity ratios D/G for Gr/Ge(001)/Si(001) samples before (**a**) and after 150 h of water vapor treatment (**b**). The data come from the maps of the Raman spectra taken in 100 points (**a**) and 150 points (**b**) (“count” means the number of points for indicated range of the parameters D/G).

**Figure 8:**
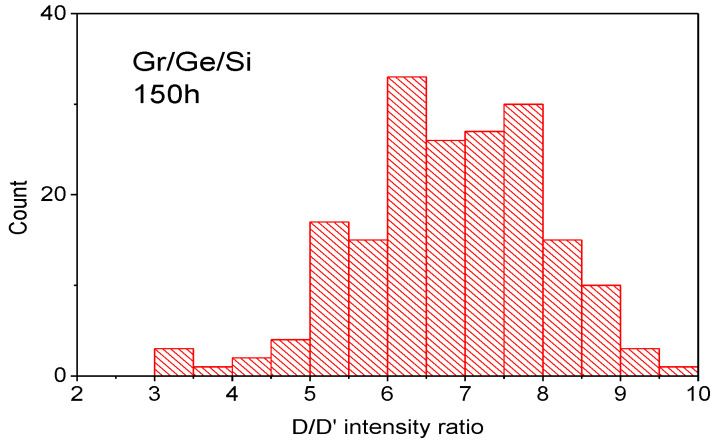
Histogram of the intensity ratio D/D’ for the Gr/Ge/Si sample after 150 h of water vapor treatment. The data come from the map of the Raman spectra taken in 150 points (“count” means the number of points for indicated range of the parameters D/D’).

The common feature of both spectra shown in Figure 6 is the D peak connected with graphene defects which has a relatively strong intensity. The ratio D/G intensity shown in the histogram in Figure 7 is about 1.5 for graphene on Ge before the water vapor treatment (virgin) and 1.9 for the sample after the water vapor treatment. This indicates that the water vapor treatment creates new defects in the graphene layer. The average concentration of defects established from the D/G ratio [20] increases from about 10^12^ cm^−2^ before, to about 1.5 × 10^12^ cm^−2^ after, the water vapor treatment. The ratio of the D/D’ intensity provides information about the nature of the defects created by the water vapor. The ratio of D/D’ modes is about eleven for the untreated sample (virgin) (measured only in a few points because of a very long collection time) and changes to about seven for the sample after 150 h of water vapor treatment, as shown in the histogram in Figure 8. This indicates that according to [20] the domination of sp^3^ defects in the virgin sample changes to domination by point defects in the water vapor treated sample. Some experimental points around a value of three to four of the D/D’ ratio (Figure 8) also indicates the presence of extended defects (boundary defects) [21]. The D and D’ modes for all the kinds of defects have the same energy, so their intensity as a summation effect gives some idea of what kind of defect dominates but do not exclude the presence of some small amounts of other kinds of defects, e.g., boundary defects in the virgin sample.

**Figure 9:**
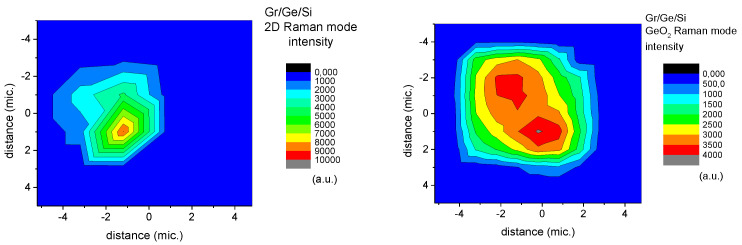
The maps of Raman intensity of the 444 cm^−1^ line due to GeO_2_ (**right** map) and the corresponding map of the 2D peak intensity (**left** map) measured on one of the blisters.

The Raman intensity of the graphene spectrum is closely related to the intensity of GeO_2_ lines. The Raman intensity maps of either the 444 cm^−1^ line connected with GeO_2_ or the 2D peak within a blister are shown in Figure 9.

**Figure 10:**
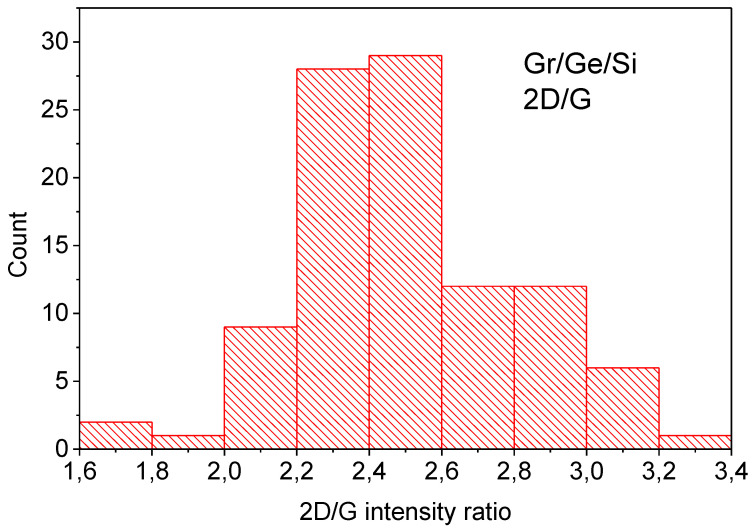
Histogram of the intensity ratio of 2D/G for the Gr/Ge/Si sample after 150 h of water vapor treatment. The data come from the map of the Raman spectra taken in 100 points (“count” means the number of points for indicated range of the parameters 2D/G).

**Figure 11:**
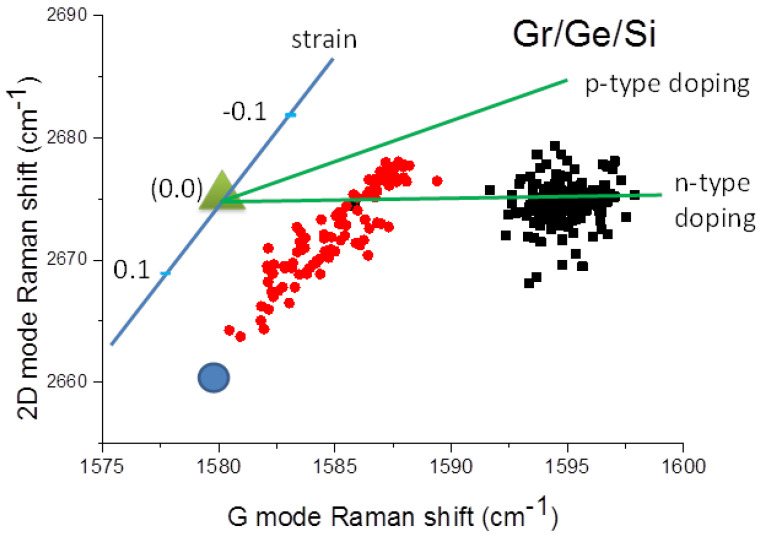
Plot of the position of the 2D band in relation to position of the G band for Gr/Ge(001)/Si(001) for virgin and for sample after 150 h of water vapor treatment. The black points represent plot before water vapor treatment of sample. Red points correspond to results on the blister after the treatment. The green triangle (0,0) represents the point of no-doped (<10^12^ cm^−2^) and no-strain graphene. The blue line represents no-doped graphene with varying values of strain (slope 2.2). The green lines represent no-strain graphene width with varying density of doping (n-type or p-type) [24]. The blue circle represents the experimental point on a blister where maximal enhancement of the Raman signal was observed.

The intensity of the Raman graphene spectra at some points on the blisters is almost two orders of magnitude stronger than outside of them (Figure 6). The enhancement factor varies from a few times to several tens of times depending on the position on the blister (Figure 10). The point of maximal enhancement of the Raman modes coincides with the maximal tensile strain of the graphene (see blue circle on Figure 11).

The 2D/G ratio is shown in the histogram in Figure 9. The intensity ratio of the 2D peak to the G one is close to 2.4 which indicates that only monolayer graphene is present. The presence of monolayer is confirmed by the average position of the 2D peak at 2675 cm^−1^ shown in Figure 11.

It is known that both Raman G and 2D peaks are sensitive to charge fluctuations and to strain due to the difference in the thermal expansion coefficients of graphene and the underlying substrate. The G peak energy is predominantly dependent on charges present on graphene due to the static and non-adiabatic effects [22] and the 2D peak energy is mainly dependent on strain fluctuation.

The contribution from the strain and charge can be deconvoluted using vector decomposition within the position of the 2D peak versus the G peak position [23]. Plots of the position of the 2D peak versus the G peak for graphene/Ge(001) sample before and after treatment with water vapor are shown in Figure 11.

Treating the Gr/Ge(001)/Si(001) sample for 150 h in the humid atmosphere results in a shift of a group of points in Figure 11 from the averaged G peak position from 1595 cm^−1^ to 1583 cm^−1^. This shift is connected with a decrease in carrier concentration of about three times, in agreement with [24]. This change in carrier concentration may be connected with a different interaction of graphene with Ge versus GeO_2_. However, one cannot exclude that water molecules on the surface of graphene have some influence on carrier concentration. The type of doping cannot be established from these measurements. The 2D peak position shown by red points in Figure 11 spreads from 2662 cm^−1^ to 2678 cm^−1^ which indicates predominantly tensile strain with some contribution of compressive strain as well. The tensile strain can be caused by a push up of the graphene layer during the growth of GeO_2._ The red points in Figure 11 are on the line with a slope of 2.2. This slope coincides with the one observed previously in strained graphene [25].

## 3. Discussion

Formation of GeO_2_ blisters on the surface of graphene/Ge(001)/Si(001) surface indicate the presence of graphene defects which are responsible for process water vapor penetration and oxidation of the germanium surface. However, it is puzzling why the oxidation of germanium under graphene takes place so effectively. Exposing pure germanium without graphene to the same oxidation conditions does not lead to formation of GeO_2_ on the surface. The question arises as to why presence of graphene on the surface of germanium speeds up the process of oxidation. The answer can be found in previous experiments connected with the investigation of the interaction of water with the graphene monolayer. Electron energy loss spectroscopy measurements on water adsorption on quasi-freestanding monolayer graphene on Pt(111) have shown that water molecules at room temperature dissociate, giving rise to C–H vibrational bands [26]. This indicates that graphene defects are sites at which dissociation of water molecules take place. Formation of C–H bonds may be linked with the following reactions: C + H_2_O --- C–H + OH, or 2C + H_2_O --- 2(C–H) + O. Most likely, O atoms or OH molecules may go through graphene defects and will interact with the germanium surface, leading to faster oxidation of Ge by O or by OH according to reaction:

Ge + 2OH --- GeO_2_ + H_2_

A similar process was reported in the case of oxidation of Cu surfaces [13,26]. It was pointed out that the presence of graphene on the surface Cu, instead of protecting it, in the long term speeds up its oxidation. It was pointed out that the presence of graphene on the Cu surface from the point of view of protection was “worse than nothing” [27]. It was even pointed out that the presence of graphene accelerates oxidation of Cu surfaces [28]. Similar processes are taking place in the case of graphene/Ge(001). Graphene instead of protecting the surface of germanium, speeds up the oxidation process. The presence of GeO_2_ underneath the graphene identified by Raman measurements, proves that oxidation of Ge in these places takes place. Most likely, extended defects connected with poor bonding between graphene sheets may be responsible for sites of dissociation of water molecules and places of penetration of OH molecules to the Ge surface.

The intensity of the Raman spectra obtained from the graphene that is on GeO_2_ compared to Ge(001), is strongly enhanced. This increase is similar to the increase in the Raman signal by surface enhanced Raman spectroscopy (SERS) by metallic nanoparticles, which is a well-known effect [29,30,31,32,33]. Due to free electrons, the metallic nano-particles are capable of concentrating and amplifying the electric near-field in the vicinities of their surfaces. The nano-particles responsible for SERS must be metallic and one can hardly consider the GeO_2_ blisters as being highly conductive. The Raman signal from the area of the blisters is about one or two orders of magnitude stronger than the one outside of this area. This increase in the graphene-related Raman bands has already been reported recently when water penetrates underneath the graphene layer and detaches it from the Ge substrate [17]. The low Raman signal from the graphene/Ge(001) surface has to be connected with some interaction between the graphene and the faceted Ge(001) surface. The vibrations of carbon atoms connected with phonon branches near the Γ and K points of the Brillouin zone of the graphene responsible for the G and 2D peaks are in-plane ones. The in-plane vibrations of the graphene plane being in direct contact with the hill-and-valley structures on the Ge(001) surface, may suffer a higher degree of “friction”. One can speculate that this may lead to a decrease in the intensity of the graphene Raman spectra. On the other hand, the oxidation of the germanium leads to graphene which is isolated from the germanium. Within the scope of this work we can state that the increase in the Raman signal for graphene/GeO_2_ seems to be connected with the isolation of the graphene by the GeO_2_ from the Ge(001) surface. The 2D vs. G diagram shown in Figure 11 indicates that graphene on GeO_2_ is predominantly under tensile strain, which is in qualitative agreement with the AFM results shown in Figure 2b. However, this strain is not uniform and changes from place to place within the blister area as is shown by the scattering of the blue points in Figure 11.

The Raman spectrum of graphene outside and inside the blister is characterized by a large D peak. The ratio of the intensity of the D to G peak being 1.5 for the virgin sample, changes to 1.9 for the water vapor treated sample as shown in Figure 7. That directly shows that the water vapor treatment generates new defects in the graphene lattice. The question which arises is what kind of defects are generated by the presence of water vapor? Information about the origin of the defects may be obtained from the D/D’ ratio. The D/D’ ratio for the virgin sample being about eleven, indicates the presence of sp^3^ defects. Apparently, the hill-and-valley nano-structures which are positioned at 90° to each other and run along the <100> directions on the Ge(001) surface create the conditions for the formation of sp^3^ bonds between some Ge surface atoms and carbon atoms from the graphene lattice. The presence of water vapor may break these sp^3^ bonds and change them into graphene point defects. The histogram presented in Figure 8 shows the maximum for D/D’ = 7 which is characteristic for point defects in the graphene lattice [21]. The density of point defects established from the D/G ratio increases from about 10^12^ cm^−2^ before, to about 1.5 × 10^12^ cm^−2^ after, the water vapor treatment. These point defects may be vacancies and may have the character of five-fold and seven-fold rings called Stole-Wales defects [34]. However, the pores connected with point defects like these are relatively small, and it is doubtful if they may be responsible for the penetration of water molecules through the graphene lattice. On the other hand, the density of the blisters established from the SEM measurements for the sample after 3 h of water vapor treatment is close to 3 × 10^8^ cm^−2^ which is several orders of magnitude lower than the density of point defects established from the Raman D/G ratio. That clearly proves that the defects responsible for the oxidation of germanium are not point defects. This is in agreement with the histogram presented in Figure 8 which shows the presence of some points for D/D’ between three and five. According to [20] the values of D/D’ in this region indicate the presence of extended boundary defects. Finally, a decrease in the graphene carrier’s concentration and tensile strain observed by us after water vapor treatment, are completely opposed to that observed in similar graphene samples after hydrogen intercalation [13]. In both cases the process of detachment of graphene takes place, but the mechanisms are completely different.

## 4. Conclusions

To conclude it has been shown that water vapor penetrates through defects in the graphene/Ge(001)/Si(001) layer and oxidizes underneath the germanium forming blisters of GeO_2_. It is proposed that graphene defects are sites of dissociation of water molecules and penetration of OH molecules or O to the Ge surface and become the origin of oxidation processes. The formation of GeO_2_ under graphene, dramatically increases the intensity of the graphene Raman spectrum. It is believed that the increase is due to the screening of graphene by GeO_2_ from the Ge(001) surface.

## Data Availability

Not applicable.

## Sample Availability

Samples of the compounds Graphene on Ge(001)/Si(001) Substrates are available from the authors.
